# Supplementary material for: Stacking Model‐Based Classifiers for Dealing With Multiple Sets of Noisy Labels
Source: Biom J. 2025 Mar 12;67(2):e70042. doi: 10.1002/bimj.70042 (PMC11898607; doi:10.1002/bimj.70042)
Supplement: Supplementary file 1 — Supporting Information [file BIMJ-67-e70042-s001.zip › density-based-ensemble-model-main/competitors_real_data/netlab-master/netlab-master/help/datwrite.htm]

Netlab Reference Manual datwrite


# datwrite

## Purpose

Write data to ascii file.

## Synopsis

```
datwrite(filename, x, t)
```

## Description

`datwrite(filename, x, t)` takes a matrix `x` of input vectors
and a matrix `t` of target vectors and writes them to an ascii
file named `filename`. The file format is as follows: the first
row contains the string `nin` followed by the number of inputs,
the second row contains the string `nout` followed by the number
of outputs, and the third row contains the string `ndata` followed
by the number of data vectors. Subsequent lines each contain one input
vector followed by one output vector, with individual values separated
by spaces.

## Example

For the XOR data set we have

```
 	nin   2
 	nout  1
 	ndata 4
 	0.000000e+00  0.000000e+00  1.000000e+00 
 	0.000000e+00  1.000000e+00  0.000000e+00 
 	1.000000e+00  0.000000e+00  0.000000e+00 
 	1.000000e+00  1.000000e+00  1.000000e+00
```

## See Also

`datread`

---

**Pages:**
Index


---

Copyright (c) Ian T Nabney (1996-9)
